# Lipid Composition, Digestion, and Absorption Differences among Neonatal Feeding Strategies: Potential Implications for Intestinal Inflammation in Preterm Infants

**DOI:** 10.3390/nu13020550

**Published:** 2021-02-08

**Authors:** Kathryn Burge, Frederico Vieira, Jeffrey Eckert, Hala Chaaban

**Affiliations:** Section of Neonatal-Perinatal Medicine, Department of Pediatrics, University of Oklahoma Health Sciences Center, 1200 North Everett Dr., Oklahoma City, OK 73104, USA; kathryn-burge@ouhsc.edu (K.B.); frederico-vieira@ouhsc.edu (F.V.); jeffrey-eckert@ouhsc.edu (J.E.)

**Keywords:** breast milk, intestinal inflammation, formula, lipids, lipase, prematurity

## Abstract

Necrotizing enterocolitis (NEC) is a significant cause of morbidity and mortality in the neonatal population. Formula feeding is among the many risk factors for developing the condition, a practice often required in the cohort most often afflicted with NEC, preterm infants. While the virtues of many bioactive components of breast milk have been extolled, the ability to digest and assimilate the nutritional components of breast milk is often overlooked. The structure of formula differs from that of breast milk, both in lipid composition and chemical configuration. In addition, formula lacks a critical digestive enzyme produced by the mammary gland, bile salt-stimulated lipase (BSSL). The gastrointestinal system of premature infants is often incapable of secreting sufficient pancreatic enzymes for fat digestion, and pasteurization of donor milk (DM) has been shown to inactivate BSSL, among other important compounds. Incompletely digested lipids may oxidize and accumulate in the distal gut. These lipid fragments are thought to induce intestinal inflammation in the neonate, potentially hastening the development of diseases such as NEC. In this review, differences in breast milk, pasteurized DM, and formula lipids are highlighted, with a focus on the ability of those lipids to be digested and subsequently absorbed by neonates, especially those born prematurely and at risk for NEC.

## 1. Introduction

Necrotizing enterocolitis (NEC) is a common gastrointestinal emergency in preterm infants (<37 weeks gestational age [GA]) [1]. Pathogenesis of this inflammatory disease is complex and incompletely understood, but the initiation of enteral feeding often directly precedes the development of NEC [2], potentially highlighting the importance of feed composition, dosing, and timing [1,3]. NEC is characterized by a multifactorial profile of risk factors, including prematurity, antibiotic use, and formula feeding [4], but a diet of human breast milk (HM) is known to be protective against NEC. An exclusively HM diet is associated with a NEC incidence six to ten times lower than that of preterm infants fed exclusively formula [5], while an exclusively human donor milk (DM) diet affords nearly an 80% reduction in risk [6]. While the nutritional and caloric composition of preterm formulas often mimic that of HM, formula feeds lack many of the bioactive components of HM thought to provide immunological and developmental benefits to the infant [7]. In addition, the digestive enzyme bile salt-stimulated lipase (BSSL), which facilitates the assimilation of milk lipids [8], is entirely absent in formula [9], resulting in a higher fecal lipid loss in preterm infants fed exclusively formula as compared with those on a mixed diet of HM and formula [10]. This impaired absorption of lipids in formula-fed infants harms the growth potential of these neonates, already requiring a growth rate double that of term infants [11]. In this review, lipid digestion is considered in term and preterm infants, with a focus on differences in dietary lipid composition and structuring, as well as absorption, among feeding strategies utilized in preterm infants. Additionally, these disparities are related to potential gastrointestinal consequences in the premature newborn, especially as it relates to intestinal inflammation and the risk for developing NEC.

## 2. Structural, Compositional, and Digestive Differences of Lipids in Neonatal Enteral Nutrition

As a rich source of bioactive compounds and nutrition, HM is widely accepted as the ideal food for infants, including for those born prematurely [12]. The lipid component of HM provides energy, and also critical fat-soluble vitamins [13]. The composition, quantity, and structure of lipids provided through available neonatal feeding strategies often differ significantly, with potentially important effects on infant health and development [14]. Furthermore, these differences among dietary strategies are not static, as the fat quantity and composition of HM, for example, changes throughout lactation [15], differs according to daily maternal diet [16], and fluctuates even during the brief interval of an individual feed [17].

Lipids of both HM and formula are structured within complex oil-in-water emulsions, referred to as milk fat globules (MFGs). Human MFGs are structurally composed of triglycerides (TGs), found in the core of the MFG, encased by a distinctive triple layer (inner single layer and outer double layer) biological membrane of primarily polar sphingolipids, phospholipids, non-polar cholesterol, and membrane proteins [13,18]. These layers, together, comprise the MFG membrane (MFGM), and differ dramatically in composition and function from those of formula. Human MFG inner membrane generally includes high levels of phosphatidylethanolamine (PE), small amounts of phosphatidylinositol (PI) and phosphatidylserine (PS), and fatty acid binding protein (FABP), while the outer bilayer is composed of phosphatidylcholine (PC), lipid rafts rich in sphingomyelin (SM) and cholesterol, and a number of antioxidant enzymes, mucins, and immunoglobulins [13,19]. In contrast, MFGs of bovine-based formula are typically surrounded by a thick layer of casein and whey proteins, with PC and limited PE from soybean lecithin added as stabilizers [20,21] after the original MFGM is lost through processing [22]. These structural differences in MFG configuration largely derive from the manufacturing processes associated with formula production, particularly homogenization, which renders uniformly sized MFGs of bovine-, goat-, or soy-based formula significantly smaller than the average diameter of MFGs found in HM [13,23,24]. Furthermore, aggregates of denatured milk proteins, a consequence of preventative microbiological heat treatment, often occur at the surface of bovine-based formula MFGs [21]. In total, these compositional differences between HM and formula MFGs lead to alterations of biophysical properties that are likely not benign, as studies have repeatedly shown lipid droplet size influences rates of lipolysis [25]. In preterm infants, in particular, TGs from MFGs in HM are hydrolyzed more rapidly than smaller, protein-coated MFGs in infant formula [26], likely due to enhanced digestive lipase access to the MFGs’ TG cores [27]. The polar phospholipid coating of human MFGs is also thought to contribute to enhanced intestinal digestion of HM lipids as compared with those of formula [28], while the phospholipid profile of bovine MFGs appears to aid gastric lipase digestion as compared with that of soy [29].

Cholesterol within the MFGM lipid rafts contributes to levels in HM much higher than those of formula [30,31]. While compensatory upregulation of de novo cholesterol synthesis has been demonstrated in several infant feeding studies in response to formula intake [32,33], plasma cholesterol concentrations and cholesterol synthesis rates, even following substantial cholesterol supplementation [34], do not always mirror those of breastfed infants [32,34]. The failure of supplementation to meet the physiological effects of HM cholesterol is likely a result of differences in bioavailability of the sources, as well as the complex regulation of cholesterol metabolism [35].

The TG core of the MFG, supplied via maternal mammary epithelial cell plasma membranes, as well as fatty acid (FA) synthesis by the mammary gland, contains upwards of 98% of the lipids present in HM [36]. While the TG content of preterm HM typically exceeds that of term HM [37], fat quantities of both sources increase developmentally from colostrum to mature milk [15]. Mothers of preterm infants often produce higher concentrations of saturated (SFA) and polyunsaturated (PUFA) fatty acids as compared with mothers of term infants [38]. Roughly 40% of the TG content of term mature HM is composed of monounsaturated FA (MUFA) [39], such as oleic acid, providing fluidity within the MFG core [40]. SFA make up approximately 35–40% of TGs, with the remainder of the TG core comprised of PUFA, largely *n*-6 [41]. In addition, preterm HM includes small amounts of medium-chain FA (MCFA), a rapid energy source due to direct absorption into the portal circulation [41]. Preterm formula often includes significantly more substantial MCFA content than HM via inclusion of coconut oil [42]. The structures of triglycerides present in human MFGs often differ from those in formula. Unlike those present in vegetable oils, formula fat blends, or other human tissues [43,44], triglycerides synthesized in the mammary gland are characterized by the preferential placement of long-chain SFA in the inner *sn*-2 position, increasing absorption of these saturates in the neonatal intestine [45], with unsaturated FAs occupying the *sn*-1 and *sn*-3 positions [46]. To best approximate the lipid composition of HM, formula most often incorporates bovine fats and also vegetable oil blends [13]. Unfortunately, while 70% of the palmitic acid of HM is esterified in the *sn*-2 position, only 45% of bovine palmitic acid, and less than 20% of vegetable oil palmitic acid, is esterified similarly [47]. These structural differences among HM and formula TGs result in absorption profiles which vary drastically depending upon the lipid source [48].

Human milk and formula contain the essential FAs, linoleic acid (LA, *n*-6), and α-linolenic acid (ALA, *n*-3), as well as their conditionally essential long-chain PUFA (LCPUFA) derivatives, arachidonic acid (AA), docosahexaenoic acid (DHA), and eicosapentaenoic acid (EPA). These LCPUFAs are well-recognized for their importance in preterm neurocognitive and visual development (e.g., [49]). LCPUFAs comprise up to 15% of the total HM TG content [18], and maternal diet accounts for substantial interindividual variability in HM LCPUFA content [50]. LCPUFA levels in DM are often insufficient to meet the needs of preterm infants as DM is frequently donated at a much later period of lactation when LCPUFA concentrations have naturally declined [15]. Importantly, up to 20% of the LCPUFA in HM is bound in phospholipids of the MFGM [51], the component of the MFG most likely to be negatively affected by temperature variations or mechanical processing inherent to milk banking. While inclusion of LA and ALA are mandated in infant formula, incorporation of DHA, EPA, and AA, though recommended, are typically not [52], resulting in a wide variety of levels of these conditionally essential FAs, depending upon the formula manufacturer [41]. Irrespective of the source, if infants cannot produce LCPUFAs endogenously from their respective precursor molecules, dietary intake is unlikely to meet the requirements of rapidly developing preterm infants [53,54]. Opinions are mixed as to whether all preterm infants can effectively synthesize LCPUFAs [55,56], as there are genetic differences in FA desaturase levels [57]. The ratio of LA to ALA, and by definition, their metabolites, appears important. For example, low *n*-3/*n*-6 ratios in HM or formula have been correlated with poor neurodevelopmental outcomes in infants [58,59]. The relative importance of the *n*-3/*n*-6 ratio to preterm infant health as compared with absolute amounts of LCPUFAs provided through the diet is currently debated [41,60].

When HM from the mother is not available, DM is often supplied as a source of neonatal nutrition. In order to reduce the risk of pathogen transmission, milk banks pasteurize DM and freeze it at −20 °C before use. The most common pasteurization process employed, Holder method (30 min at 62.5 °C), denatures many of the bioactive proteins found in HM [61], including BSSL [62], often resulting in suboptimal digestion of lipids by the neonate [63]. In addition, heat from pasteurization threatens the integrity of the donor MFGM, as phospholipids may be released from the outer lipid bilayer [64] and protein aggregates often accumulate at the surface of the MFGM [65]. The effects of phospholipid release and surface protein aggregation alter the size of the MFG, thus influencing lipase kinetics. Studies have been mixed on the effects of Holder pasteurization on the FA quantity of DM [66,67], but post-pasteurization freezing practices may disrupt the lipid structure of DM, with fat crystallization potentially puncturing the fragile MFGM [68]. While protein and lactose contents of DM are insignificantly altered with storage below freezing, concentrations of both lipids, and thus calories, decrease with increasing time at −20 °C [69]. Additionally, both pasteurization and storage at −20 °C are known to increase the risk of LCPUFA oxidation [70,71], particularly harmful in the context of DM when levels of both LCPUFA and intact antioxidants are already comparatively low [72]. In total, processing techniques employed in milk banks may contribute to the reduction in NEC protection offered by DM as compared with HM [6].

## 3. Digestion of Milk Fat Globules in Term and Preterm Infants

While enteral feeds arguably begin by 20 weeks of gestation with fetal swallowing [73], the digestive tract of infants only reaches functional maturity between three and six months of age [74]. In the weeks following birth, infants must transition from a metabolism heavily predicated on placental transfer of maternal glucose to one more inclusive of dietary lipids, ideally sourced from maternal HM [75]. Low pancreatic enzyme and bile salt concentrations during the neonatal period can present nutritional challenges [76], especially if the infant requires formula feeding, but HM provides essential digestive enzymes the infant lacks in order to increase lipid absorption in the developing neonate. Digestion and absorption of lipids are essential, as up to 50% of infant daily caloric needs are supplied via HM or formula fats [77]. In addition, efficient assimilation of these nutrients aids in proper growth and development, both of the intestine and systemically [78], as many of these lipids serve additional roles in signaling and cell structure [79]. In contrast with adults, the process of digestion and nutritional assimilation in infants, especially preterm infants, is not yet fully understood.

In neonates, digestion is initiated in the stomach due to the short transit time of an entirely liquid diet. Gastric acidity in preterm infants is reduced as compared with term infants and adults, resulting in a fasting pH of 3.2 to 3.5, a postprandial pH near neutral [80], and a microenvironment not conducive to proteolytic enzyme activation. In the stomach, pepsin and gastric lipase begin digestion of the MFG. Pepsin, due to an optimal working pH of 2, is both less active and abundant in preterm infants than in term infants or adults [75]. However, a longer period of gastric digestion provided by immature gastrointestinal motility, potentially in combination with additional undescribed proteases active in the preterm stomach [81], appears to compensate for this lower proteolytic capability [82]. Gastric lipase is capable of penetrating the complex MFGM of HM to initiate TG digestion at the MFG core [83], with high specificity for both LCPUFA and MCFA [84]. In infants, gastric lipase is highly effective as it does not require bile salt interaction nor low pH [84], and the enzyme is critical for lipid digestion, as lipases released downstream cannot similarly penetrate the MFGM [85]. While in the stomach, partial digestion of proteins and gastric lipase activity, together, cause coalescence of smaller MFGs into larger, and fewer, droplets [86]. Maternal enzymes present in HM are also active in the infant’s stomach and are likely responsible for some of the protein digestion occurring in the early stages [81]. This is especially important given the contractile activity of the neonatal stomach is not yet developmentally mature, resulting in fewer mechanical forces mixing stomach contents during digestion [87].

From the stomach, digestion continues in the small intestine, where bile and pancreatic enzymes secreted into the duodenum, as well as enzymes associated with the epithelial brush border, work to further break down MFGs. The pancreas secretes a variety of digestive enzymes into the duodenum, including trypsin, elastase, carboxypeptidases, pancreatic triglyceride lipase (PTL), phospholipase A2 (PLA2), BSSL (also known as carboxyl ester lipase [CEL] or bile salt-dependent lipase [BSDL]), and pancreatic lipase-related protein 2 (PLRP2) [83]. Concurrent with the excretion of pancreatic juice, the gallbladder releases bile salts into the intestinal lumen, but the concentration of bile salts released is extremely low as compared with an adult, often dropping below that required to properly emulsify lipids to micelles [86], especially in preterm infants [88]. While bile acid (BA) synthesis rates in premature infants are significant [89], there is thought to be a lag in development of BA secretory processes [90]. The chief neonatal primary bile acids, cholic acid and chenodeoxycholic acid [88], are predominantly conjugated to taurine, as the glycine conjugation process in the liver is not yet developmentally mature [91]. While there is passive uptake of BAs in the neonate, luminal bile salt concentration does not increase significantly until later in infancy, as active transport and enterohepatic circulation of these BAs is hampered during the neonatal period [92]. Interestingly, despite both groups harboring low levels of bile salts, preterm infants fed HM absorb lipids at a higher rate than those fed formula [93]. HM, including colostrum, does contain bile salts [94], but the extent to which these exogenous BAs contribute to lipid digestion in infants, term or preterm, is unknown [92]. The effective adsorption of pancreatic lipases to MFGs is greatly aided by the restructuring of fat into bile salt micelles.

While PTL and PLA2 are the predominant pancreatic lipases involved in lipid breakdown in the adult, low levels of these enzymes in the neonate, and especially the preterm infant, necessitate PLRP2 and BSSL bearing much of the duodenal fat digestion burden [76]. PLRP2 is capable of hydrolyzing a wide spectrum of lipids, including TGs and phospholipids, and its activity correlates negatively with luminal bile salt concentrations [95], seemingly in perfect concert with neonatal intestinal physiology. BSSL, released in low concentrations by the infant pancreas, requires primary bile salt activation to enable hydrolysis of a broad array of lipid classes [86], including LCPUFAs not hydrolyzed effectively by PLRP2 [95]. Importantly, BSSL is also secreted by the mammary gland throughout the duration of lactation, providing a critical secondary, and majority, source of the enzyme for both term and preterm infants [96]. This enzyme, capable of fully hydrolyzing TGs in vitro [97], is thought to provide much of the duodenal lipid breakdown in neonates. The physiological importance of BSSL is illustrated via its prominent representation in HM, comprising up to 2% of total protein present in HM [98]. The majority of non-esterified fatty acids (NEFA) and 2-monoacylglycerols produced through BSSL activity are emulsified into micelles for enterocyte uptake, largely in the jejunum.These lipid breakdown products are subsequently re-esterified to TGs within the intestinal epithelium, packaged into chylomicrons, and exported systemically via lymph [99]. However, very high dietary fat intake or a relative inability to digest TGs, in preterm infants especially [100], can result in the channeling of lipids to the ileum, where they are much less effectively absorbed [86]. Neither term nor preterm infants fully absorb lipids, with the former excreting up to 10%, and the latter up to 30%, of the dietary lipid load [76]. In addition, animal modeling has indicated preterm infants may struggle to repackage absorbed lipid products into chylomicrons [101], presumably resulting in lipid accumulation within the distal small intestine. However, while the preterm infant suffers from insufficient pancreatic function, immature gastrointestinal motility, and a lower intestinal surface area through which to absorb nutrients, macronutrient assimilation can be aided via the digestive enzymes present in HM.

## 4. Physiological Consequences of, and Influences on, Differential Lipid Absorption in Neonates

Compositional and structural differences of lipids among neonatal feeding strategies, combined with maturational inadequacies among prematurely born infants, may result in significant physiological consequences for preterm infants. Despite the importance of fats calorically in the infant diet, gastrointestinal developmental immaturity deems neither preterm nor term infants capable of absorbing lipids to the same degree as adults [76,102]. A number of clinical trials have documented the superior lipid absorption, often denoted by coefficient of fat absorption (CFA), of HM relative to formula, especially in preterm infants [61,72,102,103,104]. Due to required processing of DM, fresh HM is also associated with a higher CFA as compared with DM [72]. Characteristics unique to each of the neonatal feeding strategies influence the degree to which lipids are absorbed in the preterm infant, with ensuing effects on growth rate, intestinal health and maturation, and the risk for NEC development.

### 4.1. Long-Chain Polyunsaturated Fatty Acids

In addition to aiding in proper infant growth and development, LCPUFAs serve as immunomodulatory agents [105], a critically important function in the context of upregulated inflammation during the preterm neonatal period [106]. The ability to produce LCPUFA derivatives with both anti-inflammatory and immunoprotective properties in a delicate balance is essential in the complex development of the infant intestine [107]. Preterm infants forgo much of the third gestational trimester in utero, thereby relinquishing the substantial maternal transfer of LCPUFAs normally taking place during this developmental period [108]. As a result, these neonates often develop severe deficiencies of LCPUFAs, particularly DHA [102,103]. Given the demonstrated ability of *n*-3 LCPUFAs to dampen and resolve inflammation [109], including within the pro-oxidative preterm environment [110], the effects of *n*-3 supplementation on the risk of developing NEC have been widely explored. In a hypoxia mouse model of NEC, fish oil supplementation, containing both DHA and EPA, reduced the severity of intestinal lesions through a reduction in inflammatory prostaglandin and leukotriene production [111]. In premature rat pups subjected to NEC, formula supplemented with either DHA or EPA resulted in lower levels of the inflammatory transcription factor, nuclear factor kappa B (NF-κB), and prostaglandin receptor expression as compared with pups fed solely the soybean oil-based control, resulting in a reduction in intestinal inflammation [112].

However, an appreciation for balance in *n*-3 to *n*-6 LCPUFAs during the developmental fetal and neonatal periods is growing [113], with studies indicating targeted supplementation of *n*-3 without a concomitant rise in *n*-6 may have detrimental effects on intestinal development. In preterm piglets, balanced supplementation of AA and DHA resulted in an increase in intestinal villus height and smooth muscle development as compared with targeted DHA supplementation alone or the soybean oil-based control, IntraLipid^®^ [114]. Singh et al. evaluated the effects of *n*-3 LCPUFA enrichment on postnatal intestinal development utilizing the fat-1 transgenic mouse, a model for fish oil-based nutrition in premature infants. Transgenic pups accumulated increased DHA stores correlating with upregulation of cellular differentiation and protective FABP gene expression, as well as a functional decrease in intestinal permeability. In contrast, wild-type pups, a model for preterm nutritional standard of care, accumulated *n*-6 FA stores and expressed higher levels of the inflammatory genes, TLR9 (toll-like receptor 9) and CAMP (cathelicidin antimicrobial peptide) [107]. High stores of *n*-3 LCPUFA in transgenic mice, however, lowered goblet cell numbers and tight junction protein expression generally considered to be important in innate immune protection, highlighting the importance of the LCPUFA *n*-3/*n*-6 balance in postnatal development.

Additional studies, therefore, have attributed protection against NEC more generally to LCPUFAs inclusive of AA. In a rat model of NEC, LCPUFA supplementation of AA and DHA, in an *n*-3/*n*-6 ratio of 1.0:1.5, significantly reduced the incidence of disease through a reduction of inflammatory mediators in the intestine [115]. Further study in this model indicated DHA + AA, egg phospholipids (AA, DHA, and choline), and DHA alone all reduced the incidence of NEC via reductions in inflammatory mediators and TLR4 signaling [116]. In vitro work demonstrated the ability of both AA and DHA, individually, to reduce inflammatory mediator capacity and TLR4 induction in rat intestinal epithelial cells [116]. Finally, Wijendran et al. demonstrated a reduction in inflammation associated with interleukin-1β (IL-1β) induction in both adult and fetal human intestinal epithelial cells with pretreatment of DHA, but pretreatment with AA reduced inflammation solely among fetal cells [117]. Much of the protection afforded by balanced LCPUFA supplementation may be attributable to a reduction in cytokine-induced intestinal barrier dysfunction [118].

Despite promising results in rodent and in vitro models, LCPUFA supplementation in human trials has demonstrated limited and mixed results in NEC prevention [49,119,120,121]. Understanding the effects of LCPUFA supplementation on infant risk for NEC has been complicated by clinical trial procedural differences in LCPUFA composition, baseline diet, inclusion criteria, dosing schedules, and the classification of NEC as a primary or secondary outcome. A single study, thus far, has demonstrated a significant reduction in preterm infant NEC risk, utilizing formula supplemented with egg phospholipids, a source of DHA, AA, and choline [122].

### 4.2. Medium-Chain Fatty Acids

HM generally contains less than 10% MCFA [123]. Due to the recognition of poor lipid absorption in preterm infants, nearly 75% of these infants [124] are fed preterm formula with a lipid profile encompassing nearly 50% MCFAs [125], primarily to circumvent the digestive requirement of micelle formation by bile salts. Recent evidence, however, suggests this practice has little benefit on short-term infant growth [123], feeding tolerance [126], or the risk for developing NEC [126]. Compared with preterm formula consisting of largely LCPUFA [127], preterm formula high in MCFA provides no significant benefit to weight gain or nitrogen retention [127], energy expenditure or storage [127], or absorption of lipids and minerals [123,128]. Interestingly, in vitro studies have indicated the proportion of MCFAs in infant formula does not significantly influence the rate of gastrointestinal lipolysis [129]. 

The use of medium-chain triglycerides (MCTs) derived from coconut oil is a likely cause for the lack of benefit derived from MCFA supplementation of preterm formula. HM TGs containing MCFAs typically occur as one MCFA paired with two LCFAs, while coconut oil typically contains TGs with three MCFAs, with further significant differences noted in MCFA chain length and saturation levels [130]. These differences in FA stereospecificity within the TG result in equivalent release of MCFAs within the stomach when comparing formula with HM, despite significantly higher MCT levels in the former [85]. Furthermore, high levels of formula MCTs may interfere with the absorption of essential FAs, such as DHA [131].

### 4.3. Beta-Palmitate

Palmitic acid bound to the *sn*-2 position of a TG, the biochemical default of HM, is termed β-palmitate [132]. Both term and preterm infants absorb palmitic acid and calcium most effectively when the long-chain SFA is structured as β-palmitate [133]. Alternatively, when palmitic acid or other saturated LCFAs are positioned in the *sn*-1,3 positions of a TG, as with most formula [74], early lipase digestion in the duodenum paired with free calcium ions in the intestinal lumen can result in the formation of insoluble calcium soap complexes [134]. The creation of calcium soaps prompts fecal loss of precious calories, and often, calcium deficiency in the preterm infant [135]. Preterm infants are commonly administered supplemental calcium due to low stores of skeletal calcium and insufficient levels provided through preterm HM [136]. Unfortunately, the process of calcium supplementation further exacerbates the formation of calcium soaps [76,137], resulting in reduced absorption of both calcium and LCFAs, especially among preterm infants fed formula. In general, infants fed HM are characterized by higher bone mineral densities as compared with those fed formula, despite similar mineral intakes [138]. The formation of calcium soaps is thought to largely explain this disparity [133]. As palmitic acid comprises a substantial proportion of formula FAs [41], infant formulas containing low levels of palmitic acid, or palmitic acid structured primarily as β-palmitate, have been developed to reduce lipid and calcium malabsorption [135,139]. However, while positive effects on stool consistency have been noted, β-palmitate-heavy formulas have not been well-studied, and therefore are not widely utilized [140]. Importantly, the benefits of β-palmitate appear to extend beyond absorption to generalized intestinal health and development. In a mouse model of spontaneous enterocolitis, a diet high in β-palmitate as compared with a diet high in *sn*-1,3 TGs provided protection against intestinal inflammation through anti-inflammatory regulatory T cell responses and upregulation of antioxidant defenses [141]. β-palmitate also appears to positively influence the developing infant microbiome. Partial replacement of palm oil-based formula *sn*-1,3 palmitic acid with β-palmitate in term infants increased the beneficial microbiome constituents, Bifidobacteria and Lactobacillus [142], mimicking similar associations between β-palmitate and the microbiome in infants fed HM [143].

### 4.4. Bile Salt-Stimulated Lipase

Optimal neonatal digestion and absorption of lipids, especially LCPUFA [144], hinges upon exogenous BSSL provided through HM [104,145]. However, infant formula lacks BSSL entirely, while pasteurization of DM inactivates the enzyme. Malabsorption of LCFA, especially LCPUFA, and thus reduced growth [146], in preterm infants fed formula is, at least in part, attributed to the lack of this nonspecific lipase [100]. In an animal model for neonatal nutrition, kittens fed formula lacking BSSL gained half the weight of their naturally nursed littermates [147]. Weight loss was reversed, however, when the kittens were supplied with human BSSL. In preterm human infants, the inactivation of BSSL in pasteurized HM reduced weight gain and lipid absorption as compared with fresh, unprocessed HM from the same source [146]. In addition to preventing proper lipid digestion and absorption, animal modeling has indicated neonatal BSSL deficiency may induce functional intestinal damage. In neonatal mouse pups, BSSL inhibition results in undigested fats accumulating in enterocytes of the distal ileum, physically injuring the villus epithelium [14]. To address this prominent lipase deficiency in neonatal nutrition, a phase 2 clinical trial investigated the supplementation of recombinant human BSSL (rhBSSL) to preterm infants on a diet of DM or formula. Both LCPUFA absorption and growth rate increased with the addition of rhBSSL as compared with placebo [63]. However, a phase 3 trial showed improvement in preterm infant growth following rhBSSL supplementation only in a subset of small for gestational age (SGA) infants [148].

### 4.5. Complex Lipids

The unique composition and structure of the human MFG have proven difficult to mimic in infant formula [46] and are often disrupted by processing methods associated with DM [65]. Complex lipids, located both within the MFGM and in membranes of HM exosomes, comprise up to 1% of the lipids found within HM [41]. The most prevalent complex lipid within HM is the sphingolipid SM, accounting for 36% of the combined sphingolipid and phospholipid pool, with glycerophospholipids, PE and PC, each accounting for less than 30% [20,149]. In contrast, the bovine-based formula pool of complex lipids is dominated by PC, with significantly less SM [150]. As with the TG FA profile, however, the FA composition of complex lipids in HM is not static, and can be altered by maternal diet (e.g., [151]).

Sphingolipids are a significant component of the mucosal brush border. These compounds can be synthesized during growth along the crypt-villus axis [152] or taken up through the diet [153]. While importance of sphingolipids to infant health has been predicted, the roles of many of the sphingolipids within the preterm infant gut, specifically, are largely speculative, as most studies have been conducted in adults. Interestingly, the highly saturated and tightly packed nature of HM sphingolipids delays their digestion to the middle and distal intestine, exposing the distal ileum and colon, sites most commonly affected by NEC lesions, to these unique compounds [46,154]. In addition, sphingolipids have been demonstrated to accumulate in the mucosa of the small intestine at twice the rate of the colon [155], further implicating their potential importance to NEC development. Sphingolipids in HM consist principally of SM, gangliosides, glucosylceramide, and lactosylceramide [152]. Sphingomyelin in HM, often consisting of a phosphocholine head group and LCFA tails [13], is digested to the bioactive metabolites, ceramide, ceramide-1-phosphate (C1P), sphingosine-1-phosphate (S1P) [46,156], and sphingosine, the central building block of all sphingolipids [153]. Within the gut, SM and its metabolites likely play a role in trafficking of lymphocytes [157], proliferation of intestinal cells [152], angiogenesis [46], apoptosis induction [158], cholesterol uptake and lipoprotein synthesis [159], and maintenance of the intestinal barrier [46]. In rat pups, SM contributed to accelerated intestinal maturation [160] and myelination of nerve fibers [161]. In addition, when SM reaches the distal ileum and colon intact, the compound may positively influence the microbiome away from pathogenic gram-negative bacteria [162], as well as inhibit the actions of lipopolysaccharide (LPS) [163] and protect against lipid-related intestinal inflammation [164]. To date, the only study examining the addition of SM to HM did not consider gastrointestinal outcomes, but did indicate very low birthweight (VLBW) infants demonstrated improved neurobehavioral development [165].

Gangliosides, glycosphingolipids containing ceramide and sialic acid [153], may play a critical role in infant health. While found in relatively low abundance in HM [166], gangliosides are nearly absent from bovine-based formula [167]. During lactation, gangliosides are at their highest level in colostrum [168], and both the composition of the FA tails and the sialylation patterns of these molecules shift during the lactation period [46]. Dietary gangliosides are likely absorbed whole and deposited in the membranes of intestinal enterocytes, both apically and basolaterally [169], where they may influence membrane functionality through displacement of cholesterol [170]. Much like SM, gangliosides can also be synthesized endogenously along the crypt-villus axis [152]. In the intestine, gangliosides have been ascribed many important functions, including reduction of inflammatory signaling [171,172], competitive binding of pathogens [173], and modulation of the immune response [46]. In preterm infants, formula supplemented with porcine gangliosides positively influenced the microbial composition of the gut, with increased Bifidobacteria and decreased Escherichia coli counts [174]. Experiments in both rodent and ex vivo human tissues have indicated gangliosides, particularly the species most abundant in human colostrum (GD3) [175], exert protection against NEC, largely through a reduction in inflammatory signaling [176]. While individual complex lipids, such as SM or gangliosides, may have positive effects on the risk of NEC development, studies have indicated that the ratio of sphingolipids deposited or synthesized in the small intestine may also provide an early indication of the development of NEC. Rusconi et al. found an increase in the SM content and decrease in ceramide accumulation in the stools of infants immediately preceding the development of Bell’s Stage ≥ 2 NEC, potentially indicating alterations in sphingolipid metabolism may pave the way for development of NEC [177].

Recognition of the importance of intact complex lipids in both MFGMs and HM exosomes has grown. With the recent ability to isolate HM exosomes, in vitro and in vivo experiments have demonstrated their utility in the attenuation of NEC-induced intestinal damage via influences on goblet cell mucus production and generalized reduction of inflammation [178]. While exosomes transport a number of bioactive molecules, including proteins, mRNA, miRNA, and DNA [179], their membrane of complex lipids may very well contribute to the protection against NEC. Advances in manufacturing technology now allow for extraction and concentration of the MFGM from bovine milk [180]. Supplementation with bovine MFGM in infants has resulted in neurodevelopmental and cognitive improvements [181,182], as well as fewer infections [183]. Animal modeling has demonstrated additional benefits of bovine MFGM specific to the intestine, including enhanced intestinal growth and maturation [180], shifts in the microbiome composition approximating that of naturally nursed infants [180], and protection against intestinal inflammation [184]. In a neonatal rat model of NEC induced by hypoxia and hypothermia, formula-fed pups supplemented with bovine MFGM demonstrated increased weight gain, reductions in NEC incidence and mortality, and a decrease in intestinal damage as compared with pups fed formula alone [185]. Yang et al. reported that treatment of neonatal rats with a bovine milk polar lipid extraction, including both sphingolipids and phospholipids, reduced NEC symptoms through a reduction in both apoptosis and inflammation in the intestinal epithelium [186]. Though supplementation of bovine MFGM does not address the discrepancy in MFG size between infant formula and HM, the individual protein, sphingolipid, and phospholipid components of the MFGM clearly exert positive physiological influences in the postnatal environment [22].

### 4.6. Lipid Malabsorption and Intestinal Inflammation

The accumulation of incompletely digested lipids, often NEFAs, in the distal intestine of premature infants can cause inflammation and structural damage to the intestinal epithelium [187]. NEFAs are not bound to protein, and therefore, are natural detergents that are able to bind and disrupt lipophilic cell membranes. Insufficient protection of the intestinal lining by mucin, as is often seen in premature infants [188], or an extremely high concentration of NEFA accumulation, can result in intestinal necrosis [189], potentially inciting excessive intestinal inflammation and development of NEC. This phenomenon has been directly visualized in rodent models, where undigested lipids accumulated in the distal small intestine as droplets, resulting in sloughing of villus tips and compromised barrier integrity [14]. The severity of this event tracked inversely with age of the pup, indicating a period of early susceptibility in neonates [14]. Bhatia et al. noted in a rodent model of NEC that undigested lipids present in the postnatal small intestine during periods of ischemia exacerbated injury to the intestine, potentially via increased production of inflammatory mediators [190].

The source of neonatal nutrition strongly affects the likelihood of NEFA accumulation in the distal small intestine. Lipase digestion of formula, in vitro, results in 10 times the NEFA production of that of fresh HM, and these NEFA rapidly induce cell death in immune, endothelial, and intestinal epithelial cells via detergent effects on the cell membrane [191]. Importantly, as HM serves as a source of BSSL, stored HM or DM may contain higher levels of NEFA through enzymatic digestion of TGs, potentially explaining the inability of HM [5], and especially DM [6], to universally protect against NEC. Experiments, in vivo, have demonstrated predigestion of lipids in formula, resulting in hydrolyzed TGs, significantly reduced both deposition of fat droplets in the distal ileum and associated reactive oxygen species (ROS) formation as compared with standard formula feeding [187]. In addition, ROS associated with TG from standard formula feeding created malondialdehyde (MDA), a toxic and reactive product of PUFA TG peroxidation [187]. Characteristics of the dietary FA profile, such as degree of saturation or chain length, may also influence the propensity of lipids to inappropriately accumulate in the distal intestine, or alternatively, be excreted through the feces. In general, LCFAs are characterized by low infant CFA, as are highly saturated FAs [76]. Infusions of long-chain monounsaturated FAs (LCMUFAs) and MCFAs cause increased mucosal permeability and injury in newborn piglets, the severity of which positively correlates with carbon chain length and negatively correlates with age of the animal [192]. Interestingly, identical lipid infusions were physiologically benign when esterified to methyl groups [193], representing a decrease in amphiphilicity.

Malabsorbed lipids in the distal ileum of premature infants are, importantly, correlated with increased excretion of bile acids through the feces [93]. Formula intake is known to increase levels of intraluminal bile acids over that of HM consumption [194]. The immaturity of postnatal enterohepatic bile acid recycling results in significant loss of BA to the stool, particularly with formula feeding [89]. Comparisons of fecal excretion of bile salts with diet have demonstrated highest BA loss in infants fed a high LCPUFA, soy-based formula, lower loss in infants fed bovine-based formula, and lowest loss in infants fed exclusively HM [194]. Alternatively, Halpern et al. demonstrated, if high levels of bile acids are not excreted through the feces, but rather taken up by ileal enterocytes, they may be inappropriately retained within the intestinal epithelium, resulting in mucosal damage in rodents very similar to that seen in human NEC [195,196,197].

Finally, while differences in lipid intake through the diet can indirectly affect the bacterial makeup of the intestine [198], distal lipid accumulation may directly influence the microbial diversity and phylogenetic composition of the microbiome. De Wit et al. [199] demonstrated a diet high in saturated fat, resulting in lipid overflow to the distal ileum, increased representation of Firmicutes (Bacilli and Clostridia) as well as decreased microbial diversity, as measured by Simpson’s Diversity Index (SDI). Decreased microbial diversity as a consequence of undigested lipid accumulation in the distal intestine has been noted in a number of studies (e.g., [200]).

## 5. Conclusions

While differences among bioactive components of HM, DM, and infant formula are widely recognized, alterations to macronutrient structuring are less frequently discussed. The formula manufacturing process, as well as pasteurization and freeze/thaw cycles associated with sterilization and storage during milk banking, result in a number of important alterations to lipids within formula and DM as compared with fresh HM. An absence, or damage, of the MFGM, altered ratio of *n*-3 to *n*-6, varying percentage of TGs structured as β-palmitate, lack, or inactivation, of BSSL and other digestive enzymes, and a dearth of sphingolipids differentiate the digestability of formula or DM from HM. These attributes, in addition to an immature gastrointestinal system, contribute to malabsorption of ingested lipids in preterm infants. This impaired absorption could potentially affect the growth of these neonates and contribute to intestinal inflammation, hastening the development of diseases such as NEC. An avoidance of infant formula whenever possible, and potentially a move toward high hydrostatic pressure processing in association with milk banking [62], may limit lipid malabsorption and the risk for NEC in preterm infants.

## Data Availability

Not applicable.
